# Long-Chain Fatty Acids Inhibit Myeloid-Derived Suppressor Cells to Delay Tumor Progression

**DOI:** 10.3390/cimb48010118

**Published:** 2026-01-22

**Authors:** Xinyu Liu, Fanni Kong, Zhangyuzi Deng, Jing Yang, Ying Cao, Hongjie Chen

**Affiliations:** State Key Laboratory of Membrane Biology, School of Life Sciences, IDG/McGovern Institute for Brain Research, Center for Life Sciences, Peking University Third Hospital Cancer Center, Peking University, Beijing 100871, China; e1322715@u.nus.edu (X.L.); 1801111596@pku.edu.cn (F.K.); 2501110502@stu.pku.edu.cn (Z.D.); jing.yang@pku.edu.cn (J.Y.)

**Keywords:** long-chain fatty acids (LCFAs), tumor immune microenvironment, myeloid-derived suppressor cells (MDSCs), antitumor immunity

## Abstract

It is broadly realized that the body’s metabolism has a profound impact on tumor progression. However, pathophysiological mechanisms underlying the metabolic modulation of the tumor immune microenvironment remain incompletely understood. Here, we report that long-chain fatty acids (LCFAs) can directly modulate the function of myeloid-derived suppressor cells (MDSCs), a central component of establishing the tumor immune microenvironment. In vitro or in vivo exposure to LCFAs significantly reduces the expression levels of signature immunosuppressive genes of both monocytic MDSCs (M-MDSCs) and polymorphonuclear MDSCs (PMN-MDSCs). As a result, mice fed with a diet of high LCFA content exhibit delayed tumor progression and prolonged survival in different cancer models. Furthermore, this LCFA-mediated inhibition of M-MDSCs and PMN-MDSCs correlates with enhanced CD8^+^ T antitumor immunity, which is abolished in tumor-bearing nude mice. These results have revealed a previously under-recognized role of LCFAs in the tumor immune microenvironment, implicating novel therapeutic strategies for cancer treatment.

## 1. Introduction

Accumulating evidence has suggested that the metabolic status of patients can significantly influence the prognosis of various cancer types [1,2]. For instance, clinical studies have documented that long-term obesity acts as an independent risk factor for the incidence of certain cancers, and obese patients tend to exhibit worsened morbidity and mortality across many cancer types [3,4]. In addition, obesity-associated systemic inflammation impinges on the body’s antitumor immunity, thus promoting tumor progression and metastasis [5,6]. Furthermore, malnutrition, particularly in the scenario of cancer-associated cachexia, has been observed to substantially impair antitumor immunity, which may contribute to cancer-related mortality [7,8]. Therefore, an in-depth understanding of the complex crosstalk between the body’s metabolism and tumors holds the promise of more effective therapies against those dreadful human diseases.

It has long been recognized in the research field that long-chain fatty acids (LCFAs) not only function as essential metabolites for various cellular processes but also play a vital role as signaling molecules in immune responses [9]. For example, studies have demonstrated that LCFAs can engage pattern recognition receptors, e.g., Toll-like receptor 4 (TLR4), thereby initiating pro-inflammatory immune responses [10]. Also, LCFAs may trigger specific G protein-coupled receptors (GPCRs), which subsequently activate downstream signaling pathways that contribute to immunomodulatory functions [11,12]. In addition, research evidence has suggested that LCFAs exert diverse effects on central immune cell types of antitumor immunity, i.e., CD8^+^ T cells and natural killer (NK) cells [13,14]. Moreover, LCFAs may have a direct impact on the survival and resistance to apoptosis of certain cancer cell types [15]. Despite those research advances, the pathophysiological functions of LCFAs in the tumor immune microenvironment remain incompletely charted.

Myeloid-derived suppressor cells (MDSCs) are commonly induced by different tumors and represent a central component of the immune microenvironment [16,17]. Based on their cellular origin, MDSCs can be further categorized into two subtypes, i.e., monocytic MDSCs (M-MDSCs) and polymorphonuclear MDSCs (PMN-MDSCs). It has been well recognized that M-MDSCs and PMN-MDSCs both express multiple immunosuppressive signals within the tumor microenvironment, including arginase-1 (ARG1), inducible nitric oxide synthase (iNOS), programmed death-ligand 1 (PD-L1), and interleukin 10 (IL-10). Those immunosuppressive factors can collectively block the antitumor action of CD8^+^ T cells, thus facilitating tumor progression [18,19,20]. As a result, extensive research has been pursued to counteract the immunosuppressive function of MDSCs in the hope of identifying novel strategies to boost the body’s antitumor immunity [21]. Notably, previous studies reported that MDSCs became stimulated in mice by the long-term consumption of high-fat diets, leading to the exacerbation of tumor progression [22,23,24,25,26,27]. Those works reported high-fat diets as a pro-tumorigenic factor, often linked to gut dysbiosis or systemic metabolic dysfunction. For instance, a high-fat diet was shown to promote tumor growth by inducing gut microbiota-mediated leucine production, which in turn drove the differentiation of PMN-MDSCs [22]. Similarly, the source of dietary fat, particularly saturated fatty acids, could impair antitumor immunity by disrupting T cell mitochondrial function^25^. However, those findings were largely based on the long-term or chronic obesity models, and the potential effect of LCFAs on MDSCs in the tumor microenvironment under a short-term condition warrants further investigation.

## 2. Materials and Methods

### 2.1. Mouse Information

All the experimental procedures in mice were performed in compliance with the protocol approved by the Institutional Animal Care and Use Committee (IACUC) of Peking University.

Mice were maintained on the 12 h/12 h light/dark cycle (light period 7:00 a.m.–7:00 p.m.), with the normal chow diet (Synergy Pharmaceutical Bioengineering; 15.5% of calories from fat, 61.1% of calories from carbohydrates, and 23.4% of calories from proteins; LCFA content: 7.3% (*w*/*w*) of the diet) or the high-fat diet (Medicience; 60% of calories from fat, 20% of calories from carbohydrates, and 20% of calories from proteins; LCFA content: 33.6% (*w*/*w*) of the diet) and water available ad libitum unless otherwise specified. C57BL/6 wild-type and BALB/c nude mice were purchased from Charles River International (Wilmington, MA, USA). Furthermore, 6- to 8-week-old male mice were utilized for experiments.

### 2.2. Cancer Cell Cultures and Allograft Models

RM-1 prostate cancer cells (#3101MOUSCSP5290) and Lewis lung carcinoma (LLC) cells (#3101MOUSCSP5252) were purchased from the Chinese National Infrastructure of Cell Line Resource (Beijing, China) and tested negative for mycoplasma. RM-1 and LLC cells were cultured in Dulbecco’s Modified Eagle Medium (DMEM; Thermo Fisher Scientific, Waltham, MA, USA) supplemented with 10% heat-inactivated fetal bovine serum (HI-FBS; Sigma, Hong Kong, China), 100 U/mL penicillin, and 100 μg/mL streptomycin.

For the allograft models of RM-1 or LLC tumors, 2 × 10^5^ (for survival rates) or 5 × 10^5^ (for tissue analyses) cancer cells suspended in 100 μL DMEM were subcutaneously injected above the right hindlimb of each mouse. Tumor dimensions were measured every 2 days, and tumor sizes were calculated as width (mm) × width (mm) × length (mm)/2. For monitoring the survival rate, the mice were euthanized when the tumor sizes reached 2000 mm^3^.

### 2.3. Free Fatty Acid (FFA) Measurements

For the measurement of plasma FFAs, the blood was collected from the mice via intracardiac bleeding and immediately centrifuged at 3000× *g* for 10 min to obtain plasma samples. For the measurement of FFAs in tumors, tumors were freshly dissected and thoroughly homogenized on ice. The resulting homogenates were centrifuged at 5000× *g* for 10 min to clear tissue debris. FFA levels in different samples were quantified by the FFA Content Assay Kit (Solarbio, Beijing, China, #BC0595).

### 2.4. FACS Procedures

The blood was collected from the mice via intracardiac bleeding into phosphate-buffered saline (PBS) containing 5 mM Na-EDTA (pH 8.0).

The spleens were dissected and cut into small pieces on ice and then mashed through 70-μm cell strainers.

The tumors were dissected and cut into small pieces on ice. The tissues were digested in RPMI-1640 (Thermo Fisher Scientific) containing 0.1 mg/mL Liberase TL (Roche, Basel, Switzerland), 20 μg/mL DNase I (Sigma), 10 mM HEPES, and 3% HI-FBS at 37 °C for 15 min. The tissues were then mashed through 70-μm cell strainers.

The cell suspensions prepared from different mouse tissues were centrifuged at 500× *g* for 5 min and re-suspended in ammonium-chloride-potassium (ACK buffer; Thermo Fisher Scientific) to lyse red blood cells. The cell suspensions were centrifuged again at 500× *g* for 5 min and re-suspended in Hank’s Balanced Salt Solution (HBSS; Thermo Fisher Scientific) containing 3% HI-FBS. The cells were stained by intended FACS antibodies and processed on the BD LSRFortessa. FACS antibodies utilized in the experiments included panel 1 (CD45-PE (BioLegend, San Diego, CA, USA, #103106), CD11b-FITC (BioLegend, #101206), Ly6G-APC (Thermo Fisher Scientific, #17-9668-82), Ly6C-APC-Cy7 (BioLegend, #128026)); panel 2 (CD45-APC-Cy7 (BioLegend, #103116), CD3-PE (Thermo Fisher Scientific, #12-0032-82), CD4-APC (BioLegend, #100412), Foxp3-Alexa fluor 700 (BioLegend, #126422), CD8-FITC (BioLegend, #100706)); panel 3 (CD45-APC-Cy7, CD8a-eFluor 450 (Thermo Fisher Scientific, #48-0086-42), CD25-FITC (BioLegend, #101908), CD44-BV605 (BioLegend, #103047), CD69-BV711 (BioLegend, #104537), PD-1-PE-Cy7 (Thermo Fisher Scientific, #25-9985-82), TIM-3-PE (BioLegend, #119704), and LAG-3-APC (BioLegend, #125210)).

FACS data were analyzed by FlowJo (version 10.8.1, https://www.flowjo.com, accessed on 20 September 2024). Immune cell types were identified as follow: PMN-MDSCs (CD45^+^ CD11b^+^ Ly6G^+^ Ly6C^−^), M-MDSCs (CD45^+^ CD11b^+^ Ly6C^+^ Ly6G^−^), CD4^+^ T cells (CD45^+^ CD3^+^ CD8^−^ CD4^+^), Tregs (CD45^+^ CD3^+^ CD8^−^ CD4^+^ Foxp3^+^), CD8^+^ T cells (CD45^+^ CD3^+^ CD8^+^ CD4^−^). The mean fluorescence intensities of activation markers (CD25, CD44, CD69) or exhaustion markers (PD-1, TIM-3, LAG-3) on CD8^+^ T cells were quantified.

### 2.5. qPCR Analyses

The Total RNAs were extracted by the RNeasy Mini Kit (Qiagen, Hilden, Germany) and analyzed by the SYBR Green Real-Time PCR Kit (Thermo Fisher Scientific). Primers used for qPCR analyses included: *Il6* (GCTACCAAACTGGATATAATCAGGA; CCAGGTAGCTATGGTACTCCAGAA), *Il10* (CCTCTGACCCTTAAGGAGCTTAT; CGTTGCACAGGGGAGTCT), *Arg1* (AGACCACAGTCTGGCAGTTG; CCACCCAAATGACACATAGG), *Nos2* (GTTCTCAGCCCAACAATACAAGA; GTGGACGGGTCGATGTCAC), *Cd274* (GCTCCAAAGGACTTGTACGTG; TGATCTGAAGGGCAGCATTTC), and *Cyclophilin* (TGGAGAGCACCAAGACAGACA; TGCCGGAGTCGACAATGAT). *Cyclophilin* mRNA levels were utilized as the internal control.

### 2.6. In Vitro Cultures and Treatments

PMN-MDSCs and M-MDSCs in the mouse tumors of indicated conditions were FACS-stained as above and sorted on the BD FACSAria. The cells were in vitro cultured in RPMI-1640 supplemented with 10% HI-FBS, 100 U/mL penicillin, and 100 μg/mL streptomycin and then treated with 200 μM long-chain fatty acids [oleic acid (JSENB, Hong Kong, China, #UEO1383):palmitic acid (Sigma, #P5585):stearic acid (JSENB, #UE175366) = 2:2:1] for 0 h, 2 h, or 4 h. For the in vitro co-cultures, equal numbers of FACS-sorted CD8^+^ T cells and MDSCs were cultured together for 4 h before FACS analysis.

### 2.7. RNA-Seq of MDSCs

For profiling signaling pathways and transcription factors, FACS-sorted PMN-MDSCs and M-MDSCs were in vitro cultured and treated with 200 μM long-chain fatty acids (oleic acid: palmitic acid: stearic acid = 2:2:1) for 4 h. Three batches of PMN-MDSCs and M-MDSCs were subjected to paired-end RNA sequencing (RNA-seq) analyses by the Beijing Genomics Institute. The RNA-seq data were deposited to Figshare (https://figshare.com, accessed on 29 October 2025) under the link 10.6084/m9.figshare.30480146. Gene expression levels were quantified as transcripts per million (TPM). Differentially expressed genes of MDSCs after LCFAs treatments were analyzed for Gene Ontology (GO) enrichment using clusterProfiler (version 4.12.6). Gene set enrichment analysis for transcription factor motifs was carried out using enrichR against the MSigDB M3 library (https://www.gsea-msigdb.org/gsea/msigdb/mouse/collection_details.jsp, accessed on 2 October 2025). Significant results were visualized as a dot plot with ggplot2.

### 2.8. Statistical Methods

Student’s *t*-tests, ANOVA with post hoc tests, or log-rank tests were performed using GraphPad Prism (version 9.5.0, https://www.graphpad.com, accessed on 4 May 2025). All the data points represent biological replicates. Statistical details of experiments are included in the figure legends.

## 3. Results

**Figure 1:**
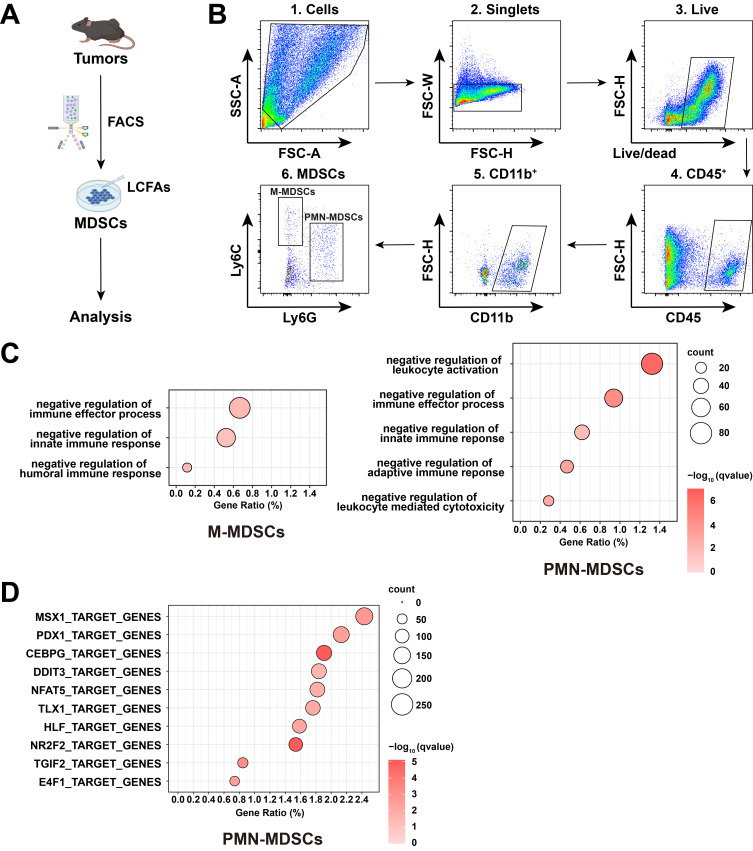
In vitro treatment of LCFAs inhibits the function of MDSCs. C57BL/6 wild-type mice were inoculated with RM-1 cancer cells, and myeloid-derived suppressor cells (MDSCs) were FACS-sorted from tumors at day 17 post-inoculation for the in vitro treatment of long-chain fatty acids (LCFAs; oleic acid:palmitic acid:stearic acid = 2:2:1). (**A**) Diagram of the experimental procedure. (**B**) Representative FACS plots of M-MDSCs and PMN-MDSCs in mouse RM-1 tumors. (**C**) Genes significantly upregulated in the control groups compared to the LCFA treatment groups of M-MDSCs or PMN-MDSCs were perform the Gene Ontology (GO) enrichment. (**D**) Differentially expressed genes between the control and LCFA treatment groups of PMN-MDSCs were analyzed for the transcription factor enrichment.

As the entry point of this study, we sought to examine the potential effect of LCFAs on in vitro cultured MDSCs. For this purpose, we inoculated C57BL/6 wild-type mice with RM-1 cells, a common allograft model of prostate tumors (Figure 1A).

**Figure 2:**
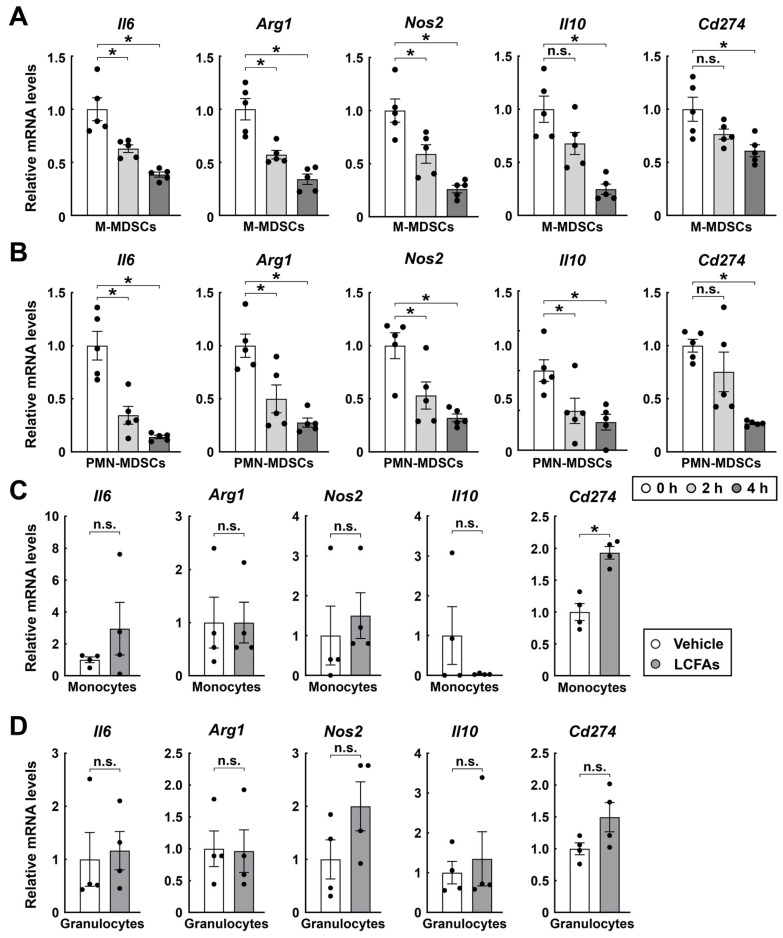
In vitro treatment of LCFAs restrains the immunosuppressive function of MDSCs. MDSCs were FACS-sorted from tumors of C57BL/6 wild-type mice inoculated with RM-1 cancer cells at day 17 post-inoculation; monocytes and granulocytes were FACS-sorted from spleens of C57BL/6 wild-type mice for in vitro LCFAs (oleic acid:palmitic acid:stearic acid = 2:2:1) treatment. (**A**,**B**) Relative mRNA levels of the signature genes of M-MDSCs (**A**) and PMN-MDSCs (**B**) treated with LCFAs for 0, 2, or 4 h. Mean ± SEM, * *p* < 0.05, n.s., not significant (one-way ANOVA test). (**C**,**D**) Relative mRNA levels of the signature genes of monocytes (**C**) and granulocytes (**D**) treated with vehicle or LCFAs for 4 h. Mean ± SEM, * *p* < 0.05, n.s., not significant (Student’s *t*-test).

M-MDSCs (CD45^+^ CD11b^+^ Ly6C^+^ Ly6G^−^) and PMN-MDSCs (CD45^+^ CD11b^+^ Ly6G^+^ Ly6C^−^) were sorted from the tumor immune microenvironment by fluorescence-activated cell sorting (FACS) (Figure 1B). In vitro cultured MDSCs were then treated with 200 μM LCFAs (oleic acid:palmitic acid:stearic acid = 2:2:1) for 4 h, and subsequent bulk RNA sequencing (RNA-seq) analysis revealed significant alterations in signaling pathways and transcription factor activity following LCFA treatment (Figure 1C,D). This transcriptional response indicates that LCFAs directly activate key regulatory networks in MDSCs, suggesting a potential mechanistic basis for LCFA-mediated modulation of MDSC function. To further validate these transcriptional changes and assess their kinetics, M-MDSCs and PMN-MDSCs were treated separately with LCFAs in vitro. Samples were collected at multiple time points (0 h, 2 h, and 4 h), and the expression of signature genes was assessed by quantitative real-time PCR (qPCR). Of importance, over time, LCFA treatment strongly reduced the expression levels of *Il6*, *Arg1*, *Nos2*, *Il10*, and *Cd274* in M-MDSCs (Figure 2A).

**Figure 3:**
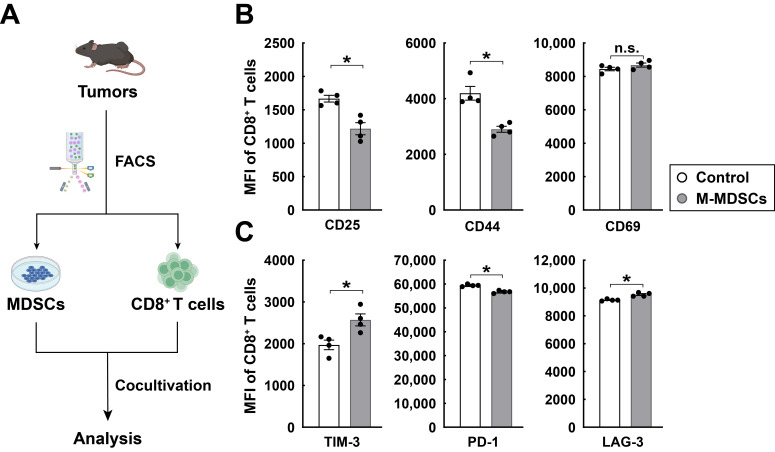
MDSCs suppress the activation of CD8^+^ T cells and induce their exhaustion during co-culture. C57BL/6 wild-type mice were inoculated with RM-1 cancer cells, and MDSCs and CD8^+^ T cells were FACS-sorted from tumors at day 17 post-inoculation for the in vitro co-culture and analysis. (**A**) Diagram of the experimental procedure. (**B**,**C**) Mean fluorescence intensity of CD8^+^ T cell activation (**B**) and exhaustion (**C**) markers after in vitro co-culture with M-MDSCs was quantified by FACS. Mean ± SEM, * *p* < 0.05, n.s., not significant (Student’s *t*-test).

This inhibitory effect of LCFAs was similarly observed in PMN-MDSCs (Figure 2B). In contrast, LCFAs had no significant impact on the expression of these immunosuppressive factors in normal monocytes and granulocytes from the spleens of untreated wild-type mice (Figure 2C,D). To further evaluate the immunosuppressive function of MDSCs, we performed an in vitro co-culture of M-MDSCs with CD8^+^ T cells at a ratio of 1:1 for 4 h. Subsequently, we measured the mean fluorescence intensity of activation and exhaustion markers on the CD8^+^ T cells (Figure 3A). As expected, M-MDSCs significantly downregulated CD8^+^ T cell activation markers such as CD25 and CD44 (Figure 3B), while simultaneously upregulating exhaustion markers, including TIM-3, PD-1, and LAG-3 (Figure 3C). These results represented that LCFAs can specifically inhibit the immunosuppressive function of MDSCs. This mechanism may be achieved by downregulating the expression of key immunosuppressive factors in MDSCs and attenuating their inhibitory effects on CD8^+^ T cells.

**Figure 4:**
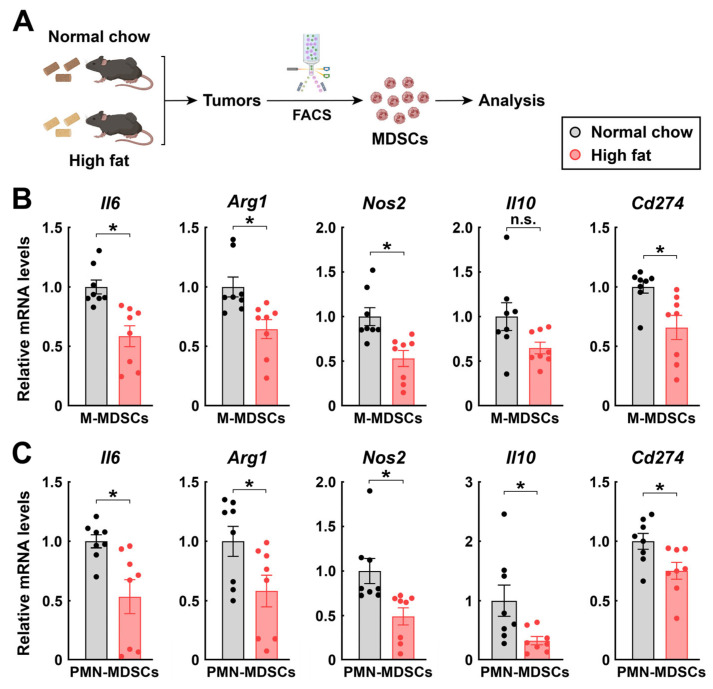
Feeding with a high-fat diet diminishes the function of MDSCs within tumors. C57BL/6 wild-type mice were fed with the normal chow diet or the high-fat diet for 3 weeks before and 17 days after RM-1 cancer cell inoculation. At day 17 post-inoculation, 2 × 10^5^ M-MDSCs or PMN-MDSCs were FACS-sorted from individual tumors for analysis. (**A**) Diagram of the experimental procedure. (**B**,**C**) Relative mRNA levels of the signature genes of M-MDSCs (**B**) and PMN-MDSCs (**C**) were quantified by qPCR. Mean ± SEM, * *p* < 0.05, n.s., not significant (Student’s *t*-test).

**Figure 5:**
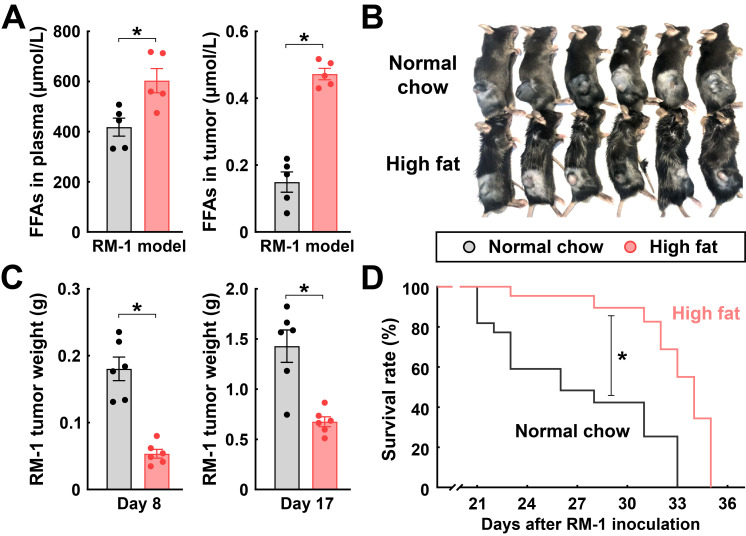
Feeding with a high-fat diet suppresses the progression of RM-1 tumors. C57BL/6 wild-type mice were maintained on the normal chow diet or the high-fat diet for 3 weeks before RM-1 cancer cell inoculation and continued with each diet condition to the indicated time points. (**A**) FFAs in the plasma or tumors of the mice were determined at 17 days post-inoculation. Mean ± SEM, * *p* < 0.05 (Student’s *t*-test). (**B**) Appearance of the tumor-bearing mice at 21 days post-inoculation. (**C**) Tumors were harvested at 8 days or 17 days post-inoculation, and tissue weights were measured. Mean ± SEM, * *p* < 0.05 (Student’s *t*-test). (**D**) Survival rates of the mice were monitored. *n* = 15 per condition, * *p* < 0.05 (log-rank test).

We sought to verify the LCFA-mediated inhibition of MDSCs in vivo. To this end, C57BL/6 wild-type mice were inoculated with RM-1 cancer cells and then fed with the normal chow diet or a diet containing a high content of fat (Figure 4A). As expected, the consumption of this high-fat diet effectively increased the levels of FFAs in the plasma and tumors of the mice (Figure 5A). MDSCs were then FACS-sorted from the tumor immune microenvironment and analyzed for their expression of signature genes. In accordance with the in vitro results above, the high-fat diet-fed mice had profoundly decreased levels of *Il6*, *Arg1*, *Nos2*, *Il10*, and *Cd274* in their M-MDSCs (Figure 4B) and PMN-MDSCs (Figure 4C).

Based on those in vitro and in vivo findings, LCFAs could mitigate the function of MDSCs in the immune microenvironment and thereby influence tumor progression. To test this possibility, we first subjected the C57BL/6 wild-type mice inoculated with RM-1 cancer cells to the normal chow or the high-fat diet. The high-fat diet-fed mice had increased FFA levels in their plasma and tumors (Figure 5A). More importantly, this high-fat diet condition was sufficient to suppress tumor growth, as tumor sizes and weights became reduced at day 8 and day 17 post-inoculation (Figure 5B,C). Accordingly, the overall survival of the high-fat diet-fed mice was prolonged compared to those on the normal chow diet (Figure 5D).

**Figure 6:**
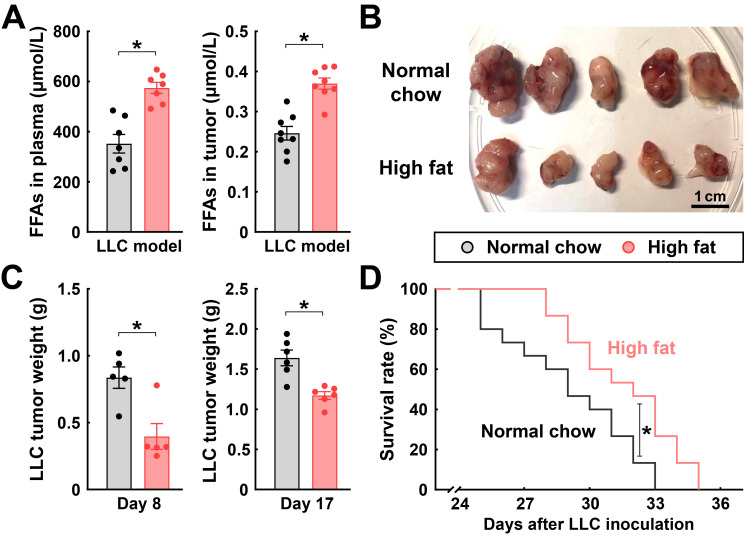
Feeding with a high-fat diet delays the progression of LLC tumors. C57BL/6 wild-type mice were maintained on the normal chow diet or the high-fat diet for 3 weeks before LLC cancer cell inoculation and continued with each diet condition to the indicated time points. (**A**) FFAs in the plasma or tumors of the mice were determined at 17 days post-inoculation. Mean ± SEM, * *p* < 0.05 (Student’s *t*-test). (**B**) Appearance of LLC tumors at 17 days post-inoculation. (**C**) Tumors were harvested at 8 days or 17 days post-inoculation, and tissue weights were measured. Mean ± SEM, * *p* < 0.05 (Student’s *t*-test). (**D**) Survival rates of the mice were monitored. *n* = 15 per condition, * *p* < 0.05 (log-rank test).

In parallel, we inoculated C57BL/6 wild-type mice with Lewis lung carcinoma (LLC), another common model of allograft tumors. The tumor-bearing mice were then fed with the normal chow or the high-fat diet. Similarly to that in the RM-1 model, the consumption of this high-fat diet boosted the FFA levels in the plasma and tumors of those mice (Figure 6A). Also, the sizes and weights of LLC tumors in the high-fat diet-fed mice were decreased at day 8 and day 17 post-inoculation compared to those on the normal chow diet (Figure 6B,C). Moreover, the survival rate of mice on the high-fat diet was improved (Figure 6D).

**Figure 7:**
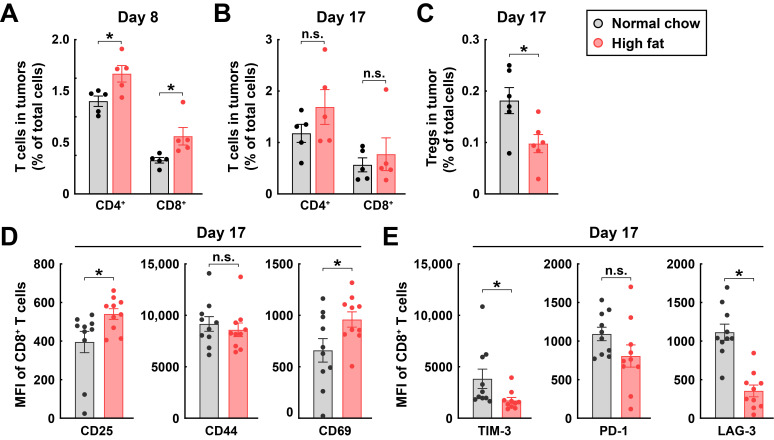
Feeding with a high-fat diet enhances T-cell infiltration in tumors. C57BL/6 wild-type mice were inoculated with RM-1 cancer cells and fed with the normal chow diet or high-fat diet. (**A**,**B**) Tumors were harvested at 8 days (**A**) or 17 days (**B**) post-inoculation. CD4^+^ and CD8^+^ T cells in tumors were quantified by FACS. Mean ± SEM, * *p* < 0.05, n.s., not significant (two-way ANOVA test). (**C**) Tumors were harvested at 17 days post-inoculation. Tregs in tumors were quantified by FACS. Mean ± SEM, * *p* < 0.05 (Student’s *t*-test). (**D**,**E**) Tumors were harvested at 17 days post-inoculation. Mean fluorescence intensity of CD8^+^ T cell activation (**D**) and exhaustion (**E**) markers was quantified by FACS. Mean ± SEM, * *p* < 0.05, n.s., not significant (Student’s *t*-test).

Consistent with the delay of tumor progression, the high-fat diet enhanced CD4^+^ and CD8^+^ T cells in the immune microenvironment at day 8 post-inoculation compared to those in the normal chow diet condition (Figure 7A). We did note that the accumulation of tumor-infiltrating T cells in the high-fat diet-fed mice tended to be normalized at a more advanced stage of tumors at day 17 post-inoculation (Figure 7B). However, we found that the high-fat diet decreased sharply regulatory T cells (Tregs) in the immune microenvironment at day 17 post-inoculation compared to those in the normal chow diet condition (Figure 7C). In further support of its specific action on the tumor microenvironment, the high-fat diet condition enhanced the CD8^+^ T cell activation markers, such as CD25 and CD69 (Figure 7D), while concurrently reducing exhaustion markers, including TIM-3 and LAG-3 (Figure 7E), in tumor-infiltrating CD8^+^ T cells at day 17 post-inoculation.

**Figure 8:**
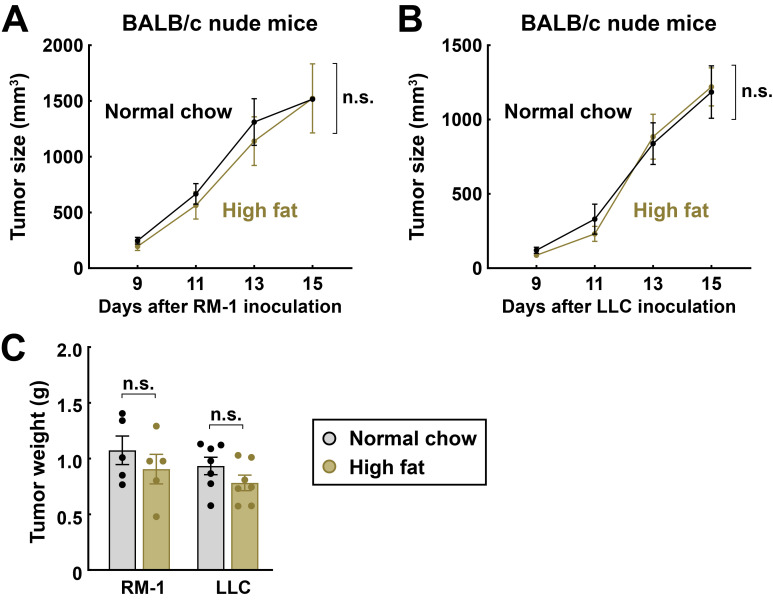
LCFA-mediated inhibition of tumor progression depends on adaptive immunity. BALB/c nude mice were maintained on the normal chow diet or the high-fat diet for 3 weeks before RM-1 or LLC cancer cell inoculation and continued with each diet condition to the indicated time points. Tumor dimensions were measured every 2 days, and tumor sizes were calculated as width (mm) × width (mm) × length (mm)/2. (**A**,**B**) Sizes of RM-1 ((**A**), *n* = 5) or LLC ((**B**), *n* = 7) tumors were monitored. Mean ± SEM, n.s., not significant (two-way ANOVA test). (**C**) Tumors were harvested at 15 days post-inoculation. Mean ± SEM, n.s., not significant (two-way ANOVA test).

Finally, we sought to verify that the LCFA-mediated inhibition of tumor progression would depend on T-cell antitumor immunity. The nude mice, a common model of deficient adaptative immunity, were inoculated with RM-1 or LLC cancer cells and then fed with the normal chow or the high-fat diet. In sharp contrast to the results obtained above in wild-type mice, the high-fat diet-fed nude mice had the growth rates of RM-1 tumors (Figure 8A) or LLC tumors (Figure 8B) comparable to those fed with the normal chow diet. Also, tissue weights of RM-1 or LLC tumors in the nude mice were not affected by the dietary conditions (Figure 8C).

## 4. Discussion

Our current study has elucidated a previously under-recognized role of LCFAs in inhibiting the immunosuppressive function of both M-MDSCs and PMN-MDSCs to delay tumor progression. We noted several reports in the field showing that MDSCs would become stimulated in the mouse models of obesity induced by the long-term consumption of high-fat diets, resulting in the acceleration of tumor progression [22,23,24,25,26,27]. For instance, a high-fat diet remodeled the gut microbiota to increase leucine production, which activated the mTORC1 pathway in myeloid progenitors and drove PMN-MDSC differentiation, thereby accelerating cancer progression [22]. Also, animal-derived dietary fats impaired antitumor immunity by inducing the accumulation of long-chain acylcarnitines, which suppressed mitochondrial function in CD8^+^ T cells [25]. In addition, the accumulation of oleic acid within the tumor microenvironment could promote tumor growth by activating macrophage signaling through the acid-sensing receptor GPR65 [26]. In contrast, our results have highlighted that short-term LCFA exposure directly inhibits the immunosuppressive function of MDSCs. While previous studies have employed chronic obesity models (≥16 weeks of a high-fat diet), in which systemic effects such as gut dysbiosis and metabolic exhaustion dominate [22,27], our study utilized an acute dietary intervention. This approach allowed us to isolate the direct, intrinsic signaling effects of LCFAs on MDSCs, prior to the establishment of chronic inflammation and secondary metabolic complications. Therefore, LCFAs may exert divergent, bidirectional effects on MDSCs as well as the overall outcome of antitumor immunity in a time-dependent manner, which warrants more in-depth investigations.

The precise molecular mechanisms by which LCFAs block the immunosuppressive function of MDSCs remain to be determined. Our findings demonstrate that treatment with LCFAs significantly reduced the expression of immunosuppressive factors in MDSCs, both in vivo and in vitro. Furthermore, this treatment increased the activation markers of CD8^+^ T cells while decreasing their exhaustion markers. Mechanistically, RNA sequencing results suggest LCFAs may primarily suppress tumor progression through the transcriptional regulation of MSX1, CEBPG, and NR2F2. Recent studies have shown that MSX1 inhibits the Notch signaling pathway, thereby inducing cell cycle arrest and apoptosis in cervical cancer cells [28]. Moreover, frequent MSX1 methylation and its interaction with PIASy have been reported to suppress angiogenesis [29]. In contrast, CEBPG has been found to inhibit apoptosis in ovarian cancer cells, thereby promoting tumor progression and directly enhancing cancer cell proliferation and migration across multiple tumor types [30,31,32]. Meanwhile, NR2F2 has been implicated in maintaining tumor stem cell-like properties and upregulating genes involved in cell migration and invasiveness, facilitating tumor cells in breaching basement membranes and surrounding tissue barriers [33]. Collectively, these findings suggest that LCFAs may exert their antitumor effects via MDSCs by upregulating MSX1-associated transcriptional pathways while concurrently downregulating those associated with CEBPG and NR2F2. Notably, previous studies have suggested that peroxisome proliferator-activated receptor gamma (PPARγ) may function as a key receptor for LCFAs in various cell types [34]. Also, transcriptional analyses have demonstrated the enriched expression of PPARγ in monocytes, neutrophils, and other myeloid cells in both mice and humans [35,36]. Therefore, it appears conceivable that the PPARγ signal may exert an essential role in designating the action of MDSCs in the tumor immune microenvironment. Indeed, several studies have demonstrated that the long-term administration of PPARγ agonists or related pharmacologic approaches could suppress tumor progression in different contexts [34,37,38]. We hypothesize that LCFAs bind to PPARγ in MDSCs, leading to the repression of immunosuppressive gene transcription and thereby restoring the antitumor function of CD8^+^ T cells. This is supported by the established role of PPARγ in antagonizing NF-κB pathways, which are critical for MDSC function [39,40].

Importantly, plasma levels of fatty acids significantly decrease in cancer patients exhibiting cachexia [41]. In light of our current work, whether such reduced availability of LCFAs may be a cause of the impairment of antitumor immunity in those patients warrants future investigation. Dietary supplementation with medium-chain triglycerides (MCTs) has been shown to counteract cachexia-related weight loss and reduce tumor size [42]. Furthermore, high-fat nutritional strategies have been explored to reverse cachexia by exploiting the metabolic differences between tumors and host tissues, thereby selectively nourishing the host at the tumor’s expense [43]. These findings are consistent with the results of our high-fat diet experiments. Moreover, whether supplementation of LCFAs could help block MDSC functions and thereby confer a beneficial effect for cancer-associated cachexia awaits clinical examinations.

The complexity of the tumor microenvironment further involves stromal components such as mesenchymal stem cells (MSCs) and their differentiation into cancer-associated adipocytes (CAAs). MSC-derived CAAs are generally thought to foster an immunosuppressive milieu and promote Treg expansion [44]. Although our current study did not explicitly evaluate the MSC-adipocyte axis, the observed antitumor effect of a short-term high-fat diet has implicated that the direct inhibition of MDSCs by LCFAs may outweigh the potential pro-tumorigenic signals from other stromal cells in our models. This is consistent with recent findings that a high-fat diet can reprogram tumor-associated macrophages, thereby significantly altering the immune landscape [45]. Future studies are needed to delineate the crosstalk between MDSCs and MSC-derived stromal cells within the tumor microenvironment.

We note that our study has certain limitations. The lipid composition in our experimental high-fat diet did not fully recapitulate the complexity of human dietary compositions, and as a result, whether the observation in mouse models may be translated into clinical applications requires careful consideration of the temporal dynamics of lipid exposure. Future investigations exploring a “precision nutrition” approach, utilizing short-term, controlled lipid exposures to modulate peripheral and intratumoral MDSC phenotypes while mitigating the risks associated with chronic metabolic dysregulation, can offer more valuable insights. Meanwhile, defining the tumor-type-specific lipidomic signatures that designate MDSC responses will be essential for potential metabolic interventions in cancer patients.

## Data Availability

The raw data supporting the conclusions of this article will be made available by the authors on request.
